# A national perspective on cardiovascular diseases in Saudi Arabia

**DOI:** 10.1186/s12872-024-03845-8

**Published:** 2024-03-27

**Authors:** Bader A. Alqahtani, Aqeel M. Alenazi

**Affiliations:** 1 Department of Health and Rehabilitation Sciences, College of Applied Medical Sciences, Prince Sattam Bin Abdulaziz University, Al-Kharj, 11942 Kingdom of Saudi Arabia; 2 Department of Health and Rehabilitation Sciences, Prince Sattam Bin Abdulaziz University, Al-Kharj, 11942 Kingdom of Saudi Arabia

**Keywords:** Cardiovascular, Elevated blood glucose, Saudi Arabia, Prevalence

## Abstract

**Background:**

Cardiovascular diseases (CVDs) are common chronic conditions that lead to morbidity and mortality worldwide. However, there are no recent national or regional reports about CVDs in Saudi Arabia. Therefore, this study aimed to estimate the national and regional prevalence rates of CVDs among the Saudi population.

**Methods:**

This study used data from an ongoing household health survey conducted by the General Authority for Statistics in 2017. The survey sample comprised 24,012 homes that were determined to be a representative sample of the population and dispersed throughout the 13 administrative areas. A self-reported diagnosis of CVD was collected by asking subjects if they had been diagnosed by a physician.

**Results:**

The prevalence of CVDs among the Saudi population aged 15 years and older was 1.6% (*n* = 236,815). The prevalence is higher in males at 1.9% compared to females at 1.4%. Age is a significant factor, with a gradual increase in CVD prevalence until the age of 50, followed by a sharp rise. The prevalence among the age group (≥ 65 years) was the highest, recording 11% (*n* = 93,971), followed by the age group (60–64 years) which reached 6.5% (*n* = 31156.71), and the lowest prevalence was found in the age group (< 40 years) as 1.2% (*n* = 108,226). When considering regional differences, Makkah has the highest prevalence at 1.9% (*n* = 85,814), followed by Riyadh at 1.7% (*n* = 79,191). Conversely, Najran has the lowest prevalence at 0.76% (*n* = 332), with the Northern Border Region having the second lowest rate at 1,46% ( *n* = 4218) These findings underscore the importance of considering both demographic and regional factors in addressing and managing cardiovascular health in Saudi Arabia.

**Conclusion:**

This study provides the most recent estimates of the national and regional prevalence rates of CVDs in Saudi Arabia. The findings suggest that CVDs are more common among older adults, males, and residents of the Makkah region. This information can be used to inform public health policies and interventions to reduce the burden of CVDs in Saudi Arabia.

## Introduction

Cardiovascular diseases (CVDs) are common chronic conditions leading to morbidity and mortality worldwide [1, 2]. Previous evidence has estimated that approximately 17.7% of 55 million deaths occurred in 2017 were attributed to CVDs [3, 4]. These diseases included modifiable risk factors such as metabolic syndrome with hypertension as the predominant risk factor accounted for 70% of CVDs and deaths. Other CVD risk factors including dyslipidemia, and diabetes are associated with myocardial infarction and stroke globally [5–7]. The impact of CVD affects all regions and nations including high, middle, and low-income countries.

Many factors including urbanization, lifestyle changes, and socioeconomic status have increased the risk of CVDs in many areas including Saudi Arabia [8]. The rapid development of the economy and oil discovery have increased socioeconomic growth. These changes spread out to lifestyle such as poor diet, lack of physical activity, and sedentary behavior leading to increased risk of diseases. Many chronic conditions have increased prevalence in Saudi Arabia recently including diabetes, hypertension, CVDs, stroke incidence, osteoarthritis, and frailty [9–16]. 

A previous systematic review reported a 5.5% prevalence of CVDs in the Gulf Cooperation Council countries [11]. However, the prevalence of CVDs was related to many other conditions and associated risk factors such as coronary heart disease, stroke, or associated factors as the main outcomes. According to the World Health Organization, CVDs include coronary heart diseases, cerebrovascular diseases, peripheral arterial diseases, rheumatic heart diseases, congenital heart diseases, deep vein thrombosis, and pulmonary embolism [17]. Previous research is limited to specific CVD types and limited samples to specific regions with limited generalizability of findings. Therefore, it is crucial to examine CVD prevalence in a comprehensive form to establish effective preventive strategies in Saudi Arabia. Thus, this study aims to examine the prevalence of CVDs in the Saudi population using national-level data across all regions in Saudi Arabia.

## Materials and methods

As part of a comprehensive Kingdom-wide screening that includes this study, the General Authority for Statistics (GASTAT). carried out a continuous household health survey A sample of 24,012 homes that is representative of the survey population and evenly distributed across the Kingdom’s administrative areas served as the basis for selecting the survey sample.

Two phases were used in the procedure for choosing sample units from the statistical structures that included the target population. In the first stage, the primary sampling units were determined. These sampling units were the enumeration regions, which were a component of the enumeration and coding phase for buildings and residential property.

**Fig. 1:**
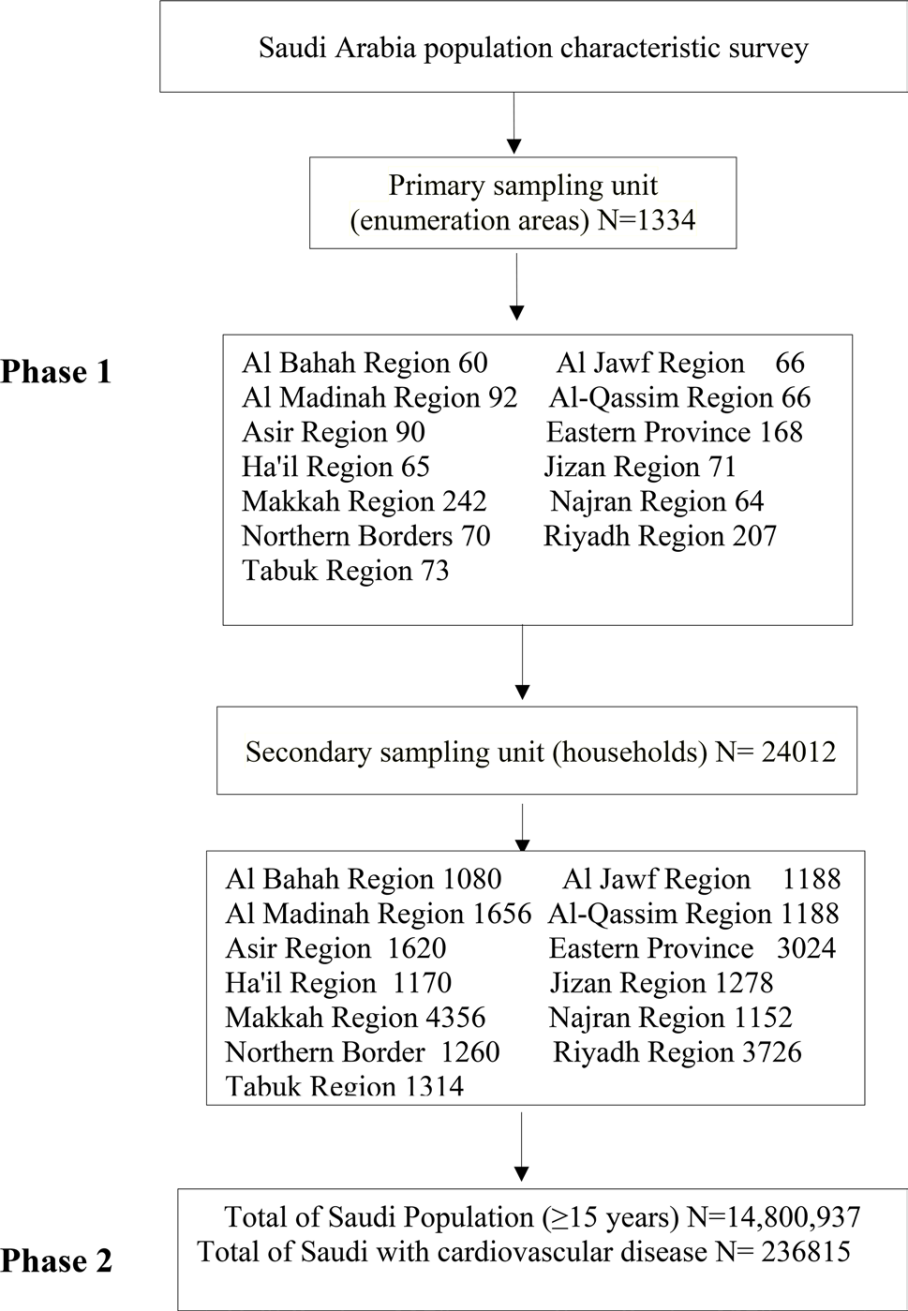
The flowchart of the survey sample selection

A total of 1334 enumeration areas were selected from among all administrative regions by using a proportional-size method and weighing the total number of Saudi homes. The final sampling units were drawn at random from the statistical areas in the second phase. By that point, the households in the first phase’s enumeration zones had been chosen by regular random sampling, yielding a total of 24,012 households throughout the Kingdom, as shown in Fig. 1. A trained field researcher from the GASTAT conducted interviews with each head of household to electronically capture all the information on an iPad system. Further elaboration on the methodology is provided elsewhere for a more comprehensive understanding [18]. 

Only patients with a confirmed diagnosis of cardiovascular diseases who had undergone the required testing and been made aware of it by a specialist doctor were included in the study.

### Sample size

The process of selecting the Primary Sampling Units (PSU) from the primary sample framework begins when the ideal home survey sample size for each administrative region has been established. The main sample frame of the household surveys’ counting areas was (1334). Using a manner appropriate for their size, they were allocated throughout all categories in every location as indicated in Fig. 1.

### Statistical analysis

Stata version 15.1 (Stata Corp, College Station, TX) was used to analyze the data, and a web-based map tool (SimpleMaps.com, Pareto Software, LLC, USA) was used to create prevalence thematic mapping. Prevalence rates (%) of CVD diagnoses (basic descriptive epidemiology) were computed for the complete sample for the current study. The prevalence rates have also been determined for the age-, gender-, and administrative region-stratified subsamples to provide insights into age, gender, and geographic variability.

## Results

**Fig. 2:**
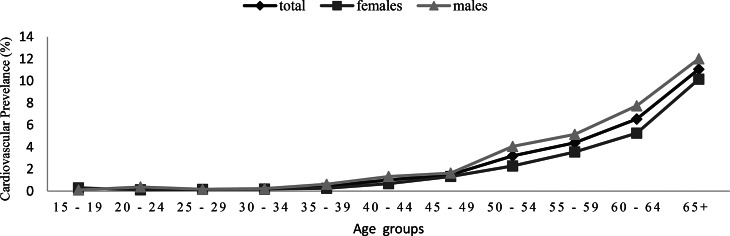
Cardiovascular disease prevalence across gender and age groups

**Fig. 3:**
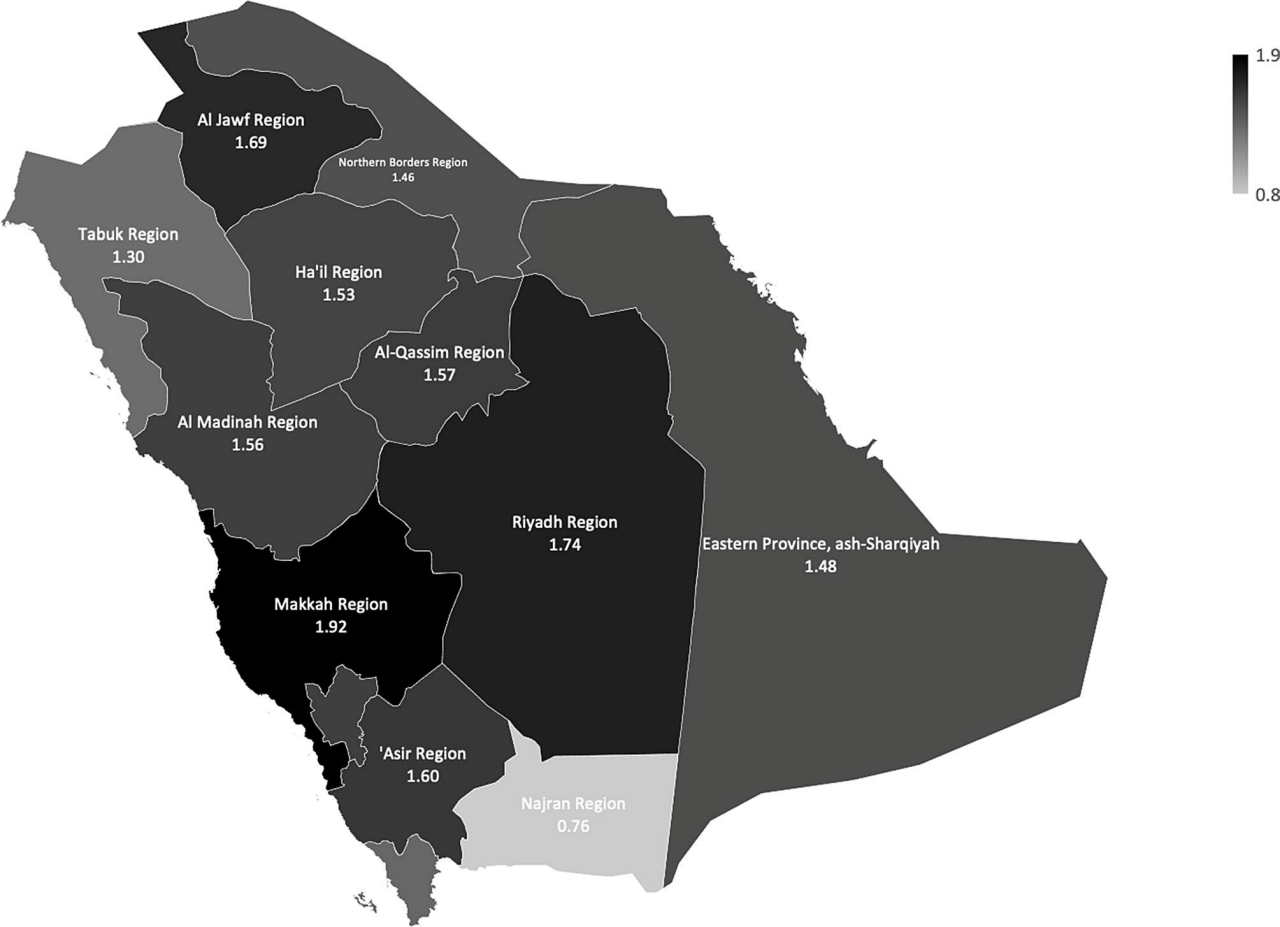
The regional prevalence rates of cardiovascular disease by administrative region

Figure 2 illustrates the percentage of the Saudi population (15 years and over) who are diagnosed with cardiovascular disorders according to gender and age groups. The proportion of the Saudi population diagnosed with cardiovascular reached 1.6% (*n* = 236,815),among the population of the Kingdom (15 years and over), and the percentage rose between the male population to reach (1.9%) (*n* = 138,491), while the female population was (1.4%) (*n* = 98,321) Table 1. The prevalence of CVD significantly increases with age, it climbs gradually until the age of 50, at which point it rises sharply. The prevalence among the age group (≥ 65 years) was the highest, recording 11% (*n* = 93,971), followed by the age group (60–64 years) which reached 6.5% (*n* = 31156.71), and the lowest prevalence was found in the age group (< 40 years) as 1.2% (*n* = 108,226). Figure 3 shows the population and the Saudi population (15 years and above) who suffer from diagnosed cardiovascular diseases in the administrative region. The figure also shows that there are significant regional differences in the prevalence rates of diagnosed cardiovascular diseases, with Makkah region having the highest prevalence 1.9% (*n* = 85,814) cases, followed by Riyadh region 1.7% (*n* = 79,191) cases, among the population of the region, while Najran region has the lowest prevalence rate of diagnosed CVD among Saudis 0.76% (*n* = 332) cases of the total Saudi population in the region, followed by Northern Border Region as the second lowest rate 1,46% ( *n* = 4218) cases of the total Saudi population in the region.

**Table 1:** Percentage of Saudi population (15 years and above) who suffer from CVD across gender and administrative regions

Aministrative Area	Female (%)	Male (%)
Al-Riyadh	1.3	2.1
Makkah Al-Mokarramah	1.6	2.2
Al-Madinah Al-Monawarah	1.4	1.8
Al-Qaseem	1.2	1.9
Eastern Region	1.6	1.3
Aseer	1.4	1.8
Tabouk	0.9	1.7
Hail	1.4	1.7
Northern Borders	1.0	1.9
Jazan	1.0	1.7
Najran	0.6	0.9
Al-Baha	1.0	2.2
Al-Jouf	1.6	1.7
**Total**	**1.4**	**1.9**

## Discussion

This study examined the prevalence of CVDs using a nationally representative sample across all regions in Saudi Arabia. The overall prevalence of CVDs was 1.6% across all regions in Saudi Arabia, with 1.9% among males and 1.4 among females. This study indicates large differences in the prevalence of CVDs according to age, sex, and region in Saudi Arabia. The highest prevalence of CVDs was reported in the older age category with 65 years and older (11.1%), males, and in Makkah region (1.92%) This study was the first report at the national level representing all regions of Saudi Arabia.

There is a lack of research related to CVDs in Saudi Arabia. The prevalence of CVDs in Saudi Arabia in our study (1.6%) was much lower than the reported prevalence in previous research (from 5.5 to 13.6%) [19–23]. This could be attributed to the type of CVDs and the samples included in these studies. Al-nozha et al. reported a prevalence of coronary artery disease of 5.5% among adults aged 30 to 70 years [19]. This report included 17,232 participants across the regions. The age inclusion in this study (30 years old) was higher than the included age in our study (15 years) which might explain the discrepancy in the prevalence. Another small study found a 13.6% prevalence of deep venous thrombosis among surgical patients [20]. However, the sample of this study is different than our sample as this study included only patients who underwent surgical intervention. Al-Sheikh et al. examined the prevalence of peripheral artery disease among patients attending a primary care center [23]. This study reported the prevalence of peripheral artery disease at 11.7% among 471 patients [23]. The small sample size and including specific age groups (45 years and older) could be attributed to the high prevalence rate in this study. Although our findings are different than previous evidence, our study included a representative large sample at a national level including all regions of Saudi Arabia. Another difference is including all CVDs in one category that has not been examined in previous research that was focused on specific diseases such as coronary heart diseases and peripheral artery diseases.

At the international level, our results were inconsistent with other reports in other countries. A recent work from China found that the standardized prevalence rate of CVD was 14.7% which was higher than the prevalence rate in our sample (1.6%) [24]. In contrast, another work from the United States reported that the prevalence of CVD was 5.5%, although this percentage is higher than the Saudi population in our study (1.6%) [25]. A lower prevalence rate of coronary heart disease was reported in England (3%) [26]. These higher prevalence rates in Eastern and Western countries compared to our study could be related to different methodologies and samples and inclusion criteria.

In Saudi Arabia, cardiovascular diseases (CVDs) are influenced by various significant risk factors. Which exhibits a higher occurrence of various CVD risk factors compared to the United States and European nations [27, 28]. These risk factors encompass ischemic heart disease, hypertension, a history of stroke, smoking, diabetes mellitus, and dyslipidemia [21, 29]. The Saudi Government has, through Saudi Vision 2030 to increase life expectancy from 75 to 80 years. CVD is one of the noncommunicable diseases that the Saudi government is committed to addressing. Since these illnesses are preventable, efforts are concentrated on reducing biological and behavioral risk factors such hypertension, obesity, dyslipidemia. In order to prevent noncommunicable diseases and reap long-term advantages, younger Saudi nationals are urged to adopt healthier lifestyles. A comprehensive report has been developed by the World Bank Group and the Saudi Public Health Authority to act as a blueprint [30]. This strategic paper describes actions to reduce the negative effects of CVD on health and the economy, so an increase in life expectancy can be reached.

This study has several limitations that need to be considered in interpretation and future work. The cross-sectional design limited the selection of the sample without measuring the incidence rate. The diagnosis of CVD was based on self-reported questions that might limit those who are unaware of the disease diagnosis to be counted in our findings. Future research should examine CVD using gold-standard diagnostic tools and measures. Lack of disease information is another limitation as the reports were not specific to which type of CVD. Other risk factors related to CVD such as obesity, hypertension, and lack of physical activity were not measured. Future studies should examine CVD within the context of prevalence and associated modifiable factors at a national level in Saudi Arabia.

## Conclusion

This study reported the national and regional prevalence of CVD among Saudi Adults using a representative sample with large variations in prevalence according to age, sex, and region. Older age, males, and Makkah region had a higher prevalence of CVD. Future research should use high-quality design including gold standard measures to establish preventive strategies for CVD in Saudi Arabia.

## Data Availability

Data used in the study is available from the corresponding author on reasonable request.
